# Severity of Anosmia as an Early Symptom of COVID-19 Infection May Predict Lasting Loss of Smell

**DOI:** 10.3389/fmed.2020.582802

**Published:** 2020-11-24

**Authors:** Jerome R. Lechien, Fabrice Journe, Stephane Hans, Carlos M. Chiesa-Estomba, Vincent Mustin, Eline Beckers, Luigi A. Vaira, Giacomo De Riu, Claire Hopkins, Sven Saussez

**Affiliations:** ^1^COVID-19 Task Force of the Young-Otolaryngologists of the International Federations of Oto-rhino-laryngological Societies (YO-IFOS), Paris, France; ^2^Department of Human Anatomy and Experimental Oncology, Faculty of Medicine, UMONS Research Institute for Health Sciences and Technology, University of Mons, Mons, Belgium; ^3^Department of Otolaryngology-Head and Neck Surgery, CHU de Bruxelles, CHU Saint-Pierre, School of Medicine, Université Libre de Bruxelles, Brussels, Belgium; ^4^Department of Otolaryngology-Head and Neck Surgery, Foch Hospital, School of Medicine, UFR Simone Veil, Université Versailles Saint-Quentin-en-Yvelines (Paris Saclay University), Paris, France; ^5^Laboratory of Oncology and Experimental Surgery, Institute Jules Bordet, Université Libre de Bruxelles, Brussels, Belgium; ^6^Department of Otorhinolaryngology-Head and Neck Surgery, Hospital Universitario Donostia, San Sebastian, Spain; ^7^Department of Otorhinolaryngology-Head and Neck Surgery, Cliniques de l'Europe, Brussels, Belgium; ^8^Maxillofacial Surgery Unit, University Hospital of Sassari, Sassari, Italy; ^9^King's College, London, United Kingdom

**Keywords:** anosmia, COVID-19, recovery, neuroepithelia, objective test

## Abstract

**Introduction:** To evaluate the recovery rate of loss of smell (LOS) with objective olfactory testing in COVID-19 patients.

**Methods:** Adults with confirmed COVID-19 and self-reported sudden LOS were prospectively recruited through a public call from the University of Mons (Belgium). Epidemiological and clinical data were collected using online patient-reported outcome questionnaires. Patients benefited from objective olfactory evaluation (Sniffin-Sticks-test) and were invited to attend for repeated evaluation until scores returned to normal levels.

**Results:** From March 22 to May 22, 2020, 88 patients with sudden-onset LOS completed the evaluations. LOS developed after general symptoms in 44.6% of cases. Regarding objective evaluation, 22 patients (25.0%) recovered olfaction within 14 days following the onset of LOS. The smell function recovered between the 16th and the 70th day post-LOS in 48 patients (54.5%). At the time of final assessment at 2 months, 20.5% of patients (*N* = 18) had not achieved normal levels of olfactory function. Higher baseline severity of olfactory loss measured using Sniffin-Sticks was strongly predictive of persistent loss (*p* < 0.001).

**Conclusion:** In the first 2 months, 79.5% of patients may expect to have complete recovery of their olfactory function. The severity of olfactory loss, as detected at the first Sniffin-Sticks-test, may predict the lack of mid-term recovery.

## Introduction

As of May 28, 2020, there have been 5.7 million confirmed cases of coronavirus disease 2019 (COVID-19) globally, with 356,000 confirmed deaths. The infection may be associated with neurological complaints, including cerebrovascular disease, peripheral nervous system symptoms, neuromuscular symptoms, or loss of smell (LOS) (1–3). Prevalence of LOS appears to vary according to clinical setting, with rates as high as 70% in mild-to-moderate disease (3, 4). There is, to date, a paucity of studies prospectively evaluating recovery rates although early reports suggest encouraging self-reported improvement rates in over 80% at only 1 week follow-up (2). Clearly, there will be a surge in patients presenting to primary care, ear, nose, and throat (ENT) and neurology physicians with anosmia following the pandemic. It is essential to be able to counsel patients regarding the likelihood of recovery, and to identify those at risk of persistent LOS, such that therapeutic strategies can be targeted appropriately.

Here, we present the first report of recovery rates of LOS, evaluated with objective olfactory testing in patients prospectively followed for 2 months after diagnosis.

## Methods

### Setting

The local ethics committee approved the study protocol (IJB-0M011-3137). Electronic informed consent was obtained for each patient.

Ninety-five adults with confirmed COVID-19 and self-reported sudden LOS were prospectively recruited through a public call from the University of Mons (Belgium). The diagnosis of COVID-19 infection was based on the WHO interim guidance and was detailed in a previous study (4). Patients had mild-to-moderate COVID-19 forms, defined as patients who did not require hospitalization. No nasal endoscopy was performed following advice from the French Society of Otolaryngology-Head Neck Surgery (SFORL) regarding the contamination risk.

### Clinical Outcomes

**Figure 1:**
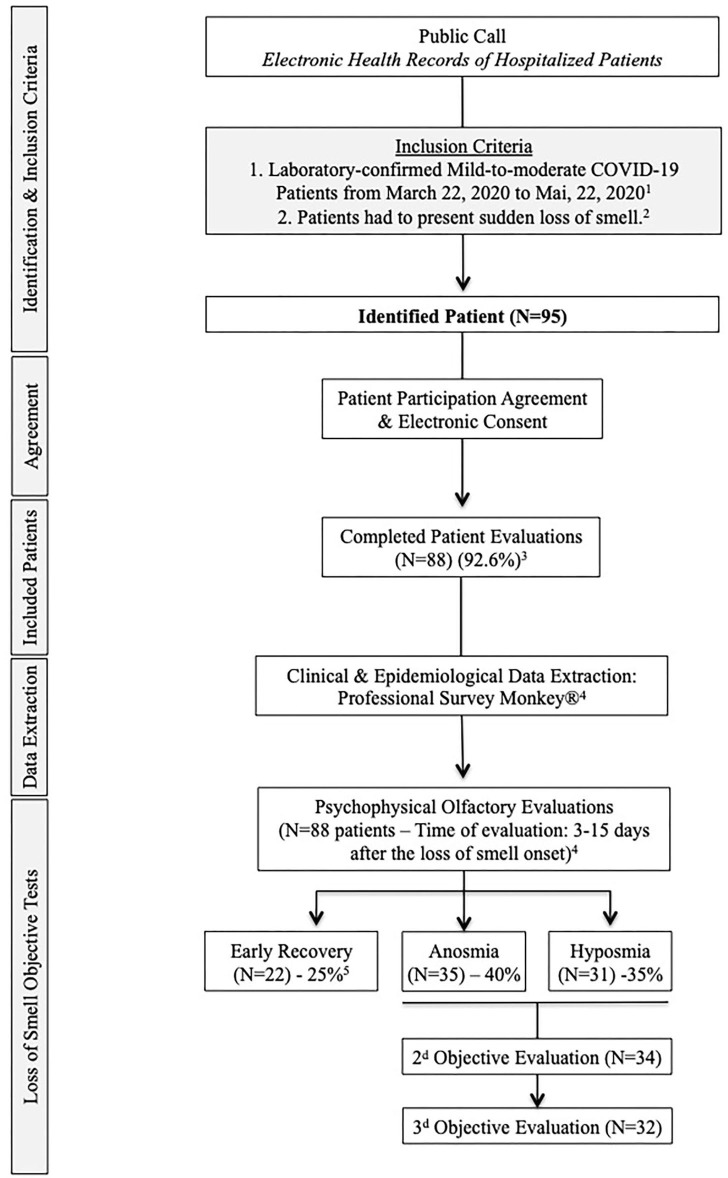
Flow Chart. 1. The included patients had mild-to-moderate COVID-19 form, defined as a disease that not required hospitalization. 2. To be included, patients had to present sudden loss of smell related to COVID-19. Patients with a history of olfactory dysfunction before the pandemic, history of nasal surgery, chronic rhinosinusitis, head and neck trauma, or degenerative neurological disease were excluded from the study. 3. Seven patients did not present to the second evaluation and were excluded. 4. The extraction of epidemiological and clinical data and the psychophysical olfactory evaluations were made at the same times (24- to 48-h maximum gap). Regarding the risk of contamination for investigators, patients were assessed when the “acute course of the disease” was resolved, corresponding to the 20 first days following the loss of smell appearance. 5. Regarding objective testing, 22 patients recovered olfaction over the 15 first days following the onset of the loss of smell.

The general and ENT symptoms were rated with a five-point scale (0 = no symptom; 4 = severe symptom). Patients completed validated patient-reported outcome questionnaires including sinonasal outcome tool-22 (SNOT-22), short version of the Questionnaire of Olfactory Disorders-Negative Statements (sQOD-NS) (4), and the smell and taste component of the National Health and Nutrition Examination Survey (NHNES) (5). Patients benefited from psychophysical olfactory evaluation using the validated 16-item Sniffin-Sticks identification test (Medisense, Groningen, Netherlands). This test compares the patient's result to normative data of young, healthy subjects, allowing categorization of patients into normosmic (16-12), hyposmic (11-9), and anosmic (8-0) (4, 6). The majority of patients had severe LOS, up to anosmia, at onset, and were invited to attend for repeated evaluation with Sniffin-Sticks until scores returned to normal levels. More details about inclusion and exclusion criteria are available in the flow chart (Figure 1).

### Statistical Analyses

Statistical analyses were performed using the Statistical Package for the Social Sciences for Windows (SPSS-v22.0; IBM Corp., Armonk, NY, USA). The evolution of olfactory outcomes was assessed through Kruskal–Wallis-test. Comparison of means was done with the *t*-test. Associations were studied with the Spearman correlation-test.

## Results

Eighty-eight patients with self-reported LOS completed the study (Figure 1). There were 59 females (67.0%), and the mean age was 42.6 ± 11.2 years. The epidemiological, clinical, and olfactory features are reported in Table 1. The most prevalent symptoms were asthenia, headache, and nasal obstruction. Dysgeusia, defined as the impairment of salty, sweet, bitter, and sour, was reported by 39.2% of patients, while 78.4% of patients reported aroma perception dysfunction. 44.6% reported that LOS developed after the general symptoms.

**Table 1:** Patient characteristics.

**Characteristics**	***N* = 88**
Age (mean ± SD), years	42.6 ± 11.2
Gender (Female/Male)	59/29
Smoker	6 (6.8)
Seasonal allergy	16 (18.2)
**Comorbidities**	
Reflux	8 (10.8)
Hypertension	5 (6.8)
Allergic rhinitis	5 (6.8)
Asthma	5 (6.8)
Diabetes	2 (2.7)
Hypothyroidism	2 (2.7)
General symptoms^*^	
Asthenia	52 (70.3)
Headache	43 (58.1)
Cough	32 (43.2)
Myalgia	32 (43.2)
Anorexia	24 (32.4)
Diarrhea	22 (29.7)
Arthralgia	22 (29.7)
Conjunctivitis	18 (24.3)
Dyspnea	17 (23.0)
Abdominal pain	14 (18.9)
Chest pain	13 (17.6)
Nausea/vomiting	11 (14.9)
Fever (>38°C)	9 (12.2)
Ear, nose, and throat symptoms^*^	
Nasal obstruction	43 (58.1)
Postnasal drip	35 (47.3)
Rhinorrhea	34 (45.9)
Taste dysfunction	29 (39.2)
Ear pain	23 (31.1)
Throat sputum	20 (27.0)
Sore throat	20 (27.0)
Dysphonia	17 (23.0)
Face pain/heaviness	16 (21.6)
Dysphagia	7 (9.5)
**SNOT-22**	33.6 ± 18.2
**sQOD-NS**	10.8 ± 5.5
Olfactory and retro-olfactory features^*^	
**Aroma sense dysfunction (retro-olfaction)**	
Total vs. partial loss of aroma perception sense	26/23
Distortion	9 (12.2)
Did not remember	3 (4.1)
No problem	39 (52.7)
**Smell dysfunction**	
Cacosmia	48 (64.9)
Phantosmia	22 (29.7)
**Onset of smell dysfunction**	
Before the other symptoms	16 (21.6)
Concomitant with other symptoms	22 (29.7)
After the other symptoms	33 (44.6)
Did not remember	3 (4.1)
**Baseline objective olfactory tests**	***N*** **= 88**
Anosmia	35 (40)
Hyposmia	31 (35)
Early recovery (1–14 days)	22 (25.0)

The percentages are given (in brackets).

* *Percentages assessed on patients who fulfilled the online NHNES questionnaire (N = 74). NHNES, National Health and Nutrition Examination Survey; SD, standard deviation; SNOT-22, sinonasal outcome tool-22*.

According to psychophysical olfactory evaluations, 22 patients (25.0%) had recovered normal olfaction within the first 2 weeks following the onset of LOS, before the realization of objective evaluations, while 9 patients (10.2%) required 15–30 days to recover. Twenty-five patients (28.4%) recovered between the 31st day and the 45th day post-LOS. Fourteen (15.9%) had olfaction recovery between the 46th day and the 60th day post-LOS (*p* < 0.001, Kruskal–Wallis-test, Appendices 1A,B). Recovery was fairly evenly distributed across the time points, such that at the time of final assessment at 2 months, 70 patients (79.5%) had achieved normal levels of olfactory function. The mean Sniffin-Sticks-test at baseline of patients who recovered within 2 months was 11.14 ± 3.22 (mean ± SD) (groups 1–4) but was significantly lowered to 5.06 ± 1.80 in patients who did not recover (*p* = 0.003, *t*-test).

There was no association between recovery and age, gender, or SNOT-22 scores. Baseline Sniffin-Sticks-test results were not significantly associated with nasal obstruction or rhinorrhea in patients who did not recover early. By contrast, we identified a positive association between postnasal drip and Sniffin-Sticks-test data in patients who had early olfactory recovery (within 2 months) (*r*_s_ = 0.29, *p* = 0.025, Spearman correlation-test). In this group (1–4), a less severe LOS was significantly associated with an earlier recovery (*r*_s_ = 0.54, *p* < 0.001).

Higher level of severity of baseline olfactory loss measured using the Sniffin-Sticks was strongly predictive of persistent loss (*p* < 0.001), as the severity of anosmia was significantly associated with the duration of such symptom (*p* = 0.002, Kruskal–Wallis-test, Appendices 1A,B). In the 35 patients who were anosmic at the first Sniffin-Sticks-test, 18 (51.4%) did not recover at 2-month post-LOS. Of those who had not recovered (group 5), the mean age was 42.3 ± 12.2 (mean ± SD) (not different from the whole population), 9 (50%) were female and 8 (44.4%) remained anosmic. This group with a persistent symptom of anosmia was not significantly associated with comorbidities.

## Discussion

To date, the short-term objective recovery rate of olfactory function is still unknown. Regarding our results, 79.5% of patients recovered normal smell sense within 2 months following the onset of LOS. We observed different patterns of recovery: some patients rapidly recovered olfaction, while in others, olfaction required more time to recover.

Typically, LOS occurring as part of the common cold is associated with nasal congestion and rhinorrhea; therefore, it may seem unsurprising to the patient, who will likely not seek medical help for many weeks until the congestion has resolved. Thus, it is conceivable that the inflammation of the nasal mucosa, including the olfactory cleft mucosa, may block the passage of the odorant molecules into the olfactory epithelium. This mechanism could be important in patients who quickly recovered olfaction, as supported by the significant association between posterior rhinorrhea and objective olfactory evaluations.

However, the lack of association between nasal symptoms and the severity of LOS in the remaining patients support the occurrence of another mechanism, which may involve neural spread of the virus through the olfactory epithelium. Nasal respiratory epithelial cells and olfactory epithelial support cells (sustentacular cells) may express moderate-to-high levels of angiotensin converting enzyme-2 (ACE2), which is the virus receptor (7). The heterogeneity in the level of ACE2 expression between individuals and cell types may support the existence of various degree of olfactory cell impairment.

The neural spread of the virus through olfactory cells is strengthened by previously described human strains of coronavirus that have also been demonstrated to invade the central nervous system and propagate to the central nervous system from within the olfactory bulb (8). Moreover, some preliminary imaging findings conducted on COVID-19 patients support the presence of olfactory bulb abnormalities [Appendices 1C,D, (9)].

Long-term follow-up studies are needed, especially for the patients with persistent olfactory loss. Currently, we believe that this group better reflects those patients typically presenting with post-viral olfactory loss in whom lower rates of spontaneous recovery have previously been reported, ranging from 32 (10) to 67% (11) in the absence of any active treatment. The identification of these patients with a higher risk of mid-to-long post-viral anosmia is important in order to commence olfactory training or systemic therapy.

The main limitation of this study is the lack of Sniffin-Sticks-test for those patients with rapid recovery. However, the realization of objective olfactory evaluation in the early course of the disease was logistically difficult regarding travel restrictions and the risk of contamination. The exclusion of these patients would lead to a selection bias, due to the lack of consideration of patients who recovered smell sense early. Moreover, we are unable to confirm that their self-reported recovery was indeed complete, as subjective ratings do not always correlate with results of psychophysical testing. Another limitation is the lack of inclusion of patients with severe COVID-19. However, it was difficult to recruit them in the early stage of the disease and their prolonged admission prohibited objective testing.

## Conclusion

In the first 2 months, 79.5% of patients may expect a complete recovery of their olfactory function. The severity of anosmia as detected at the first Sniffin-Sticks-test may predict the lack of recovery after 46 days for half of patients. Clinicians should enquire about the chemical senses in every case of COVID-19, and objective testing may help to identify those who are at risk of persistent loss and therefore benefit from olfactory training and the consideration of systemic therapy.

## Data Availability Statement

The raw data supporting the conclusions of this article will be made available by the authors, without undue reservation.

## Ethics Statement

The studies involving human participants were reviewed and approved by Institute Jules Bordet. The patients/participants provided their written informed consent to participate in this study.

## Author Contributions

JL, FJ, and SS: drafting of the manuscript. CC-E, VM, EB, CH, FJ, and SS: reviewing of the paper content, recruitment of patient, and clinical examination. FJ: statistics. All authors contributed to the article and approved the submitted version.

## Conflict of Interest

The authors declare that the research was conducted in the absence of any commercial or financial relationships that could be construed as a potential conflict of interest.

## Figures and Tables

**Appendix 1:**
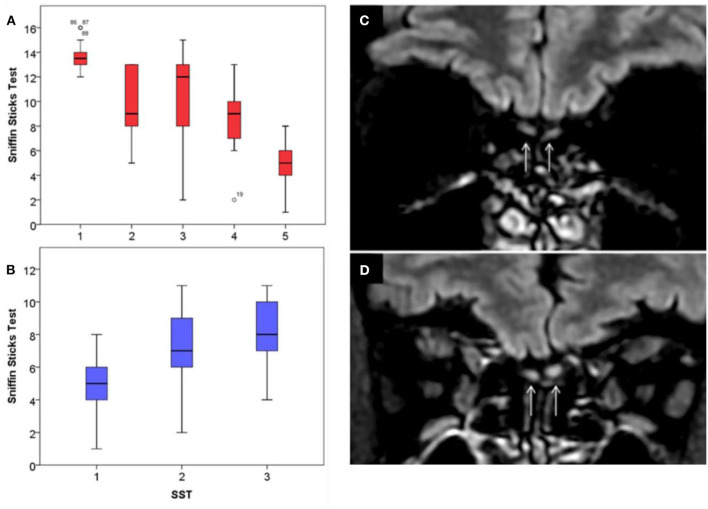
Sniffin-Sticks-test features and imaging of olfactory bulb. The first Sniffin-Sticks-tests (SST) of COVID-19 patients are represented in **(A)**. The median value of SST was presented regarding the following patient recovery subgroups: 1 (recovery: 1–14 days), 2 (15–30 days), 3 (31–45 days), 4 (45–60 days), and 5 (no recovery). Patients of group 5 had a lower baseline SST compared with other groups (*p* < 0.001). The mean value of SST of patients of group 5 significantly improved throughout the first 2 months following the onset of anosmia, represented in **(B)**. Comparison of T2/FLAIR coronal views centered on the olfactory bulbs showed normal signal in a normosmic **(C)** and T2/FLAIR hyperintensity of the olfactory bulbs in a patient with anosmia **(D)**.
